# The biohybrid autonomous robots (BAR): a feasibility of implementation

**DOI:** 10.3389/frobt.2025.1695262

**Published:** 2025-10-28

**Authors:** Georgiy N. Kuplinov

**Affiliations:** RUDN University, Moscow, Russia

**Keywords:** biohybrid robotics, batteries, muscles, biological muscles, energy capacity, autonomous robots, BAR

## Abstract

Limited battery capacity poses a challenge for autonomous robots. We believe that instead of relying solely on electric motors and batteries, basically Conventional Autonomous Robots (CAR), one way to address this challenge may be to develop Biohybrid Autonomous Robots (BAR), based on current achievements of the field of biohybrid robotics. The BAR approach is based on the facts that fat store high amount of energy, that biological muscles generate decent force per unit of cross-sectional area and that biological muscles have capability for regeneration and adaptation compared to electric motors. To reach conclusions about the feasibility of BAR, this study uses data from the fields of muscle energetics, robotics, engineering, physiology, biomechanics and others to perform analysis and interdisciplinary calculations. Our calculations show that the BAR approach is up to 5.1 times more efficient in terms of the mass of energy substrate to useful energy transported than the Conventional Autonomous Robots (CAR) with mass-produced batteries in an ideal scenario. The study also presents the model for determining the point of the rational use of the BAR, taking into the account basal metabolism of living systems. The results of this study provide a preliminary basis for further research of the BAR, putting it into the context of the other possible solutions for energy autonomy problem: Generator-Powered Autonomous Robots (GPAR) and Fuell-Cell Autonomous Robots (FCAR).

## 1 Introduction

The development of autonomous robots is hindered by a number of challenges, including the issue of energy autonomy (Abdelfetah et al., 2022). Biohybrid Autonomous Robots (BAR) are a potential candidate to solve energy problem, due to their use of glucose (Sahiin, 1990) and fat (Hanson and Hakimi, 2008; Bjorntorp, 1991) as energy sources and their ability to perform movements, as demonstrated in previous studies in the field of biohybrid robotics (Morimoto et al., 2020; Ricotti and Menciassi, 2012; Morimoto et al., 2018). Fat and glucose have energy capacities over 6,000 kcal/L (6,978 Wh/L) and 4,000 kcal/kg (4,652 Wh/kg). For comparison, the energy capacity of the most efficient Li-ion batteries is around 1,421 kcal/L (1,653 Wh/L) and 611 kcal/kg (711 Wh/kg), whereas the capacity of current mass-produced batteries is approximately half this value (Unites, 2005; Li et al., 2024). However, it is unclear whether the BAR has any advantages over the Conventional Autonomous Robot (CAR) electric battery-motor model without further analysis. This is due to the lower efficiency of biological muscles (approximately 20%) (Smith et al., 2005) compared to electric motors (approximately 83%) (Demirel, 2018), as well as other practical challenges associated with the BAR. Nevertheless, our calculations indicate that, in terms of the mass of energy substrate to useful energy transported, the Biohybrid Autonomous Robots (BAR) are up to 5.1 times more efficient than the Conventional Autonomous robots (CAR), if we omit the metabolism of living systems. If we account for the basal metabolism of the BAR, the fat-powered BAR still has an advantage compared to Conventional Autonomous Robots (CAR) from a reasonable amount of work performed. Furthermore, biological muscles demonstrate decent force and power properties per 1 cm^2^ of cross-sectional area according to our calculations and other sources.

Although we explained the rationale behind the idea, we need to provide a more detailed explanation of the concept. The primary objectives of BAR are to preserve the properties of biological muscles and to enable performing controlled biological muscle contractions.

**FIGURE 1:**
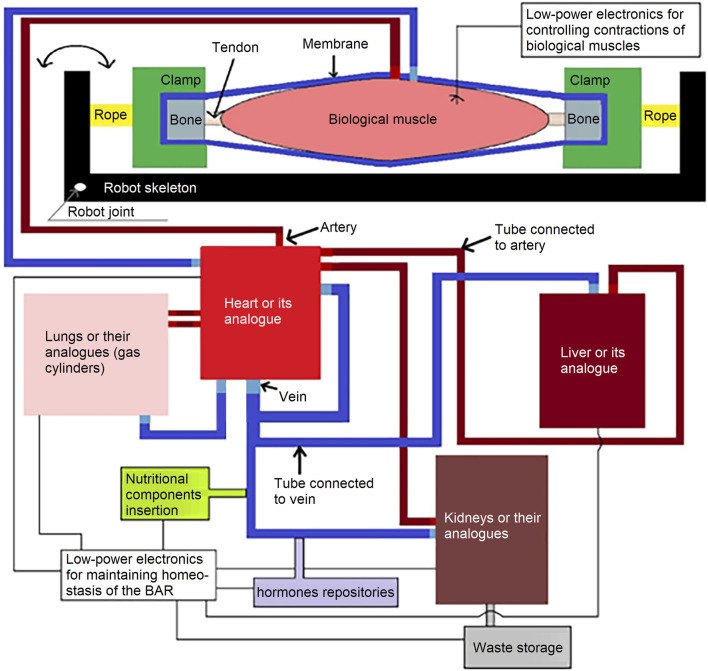
A rough draft of the BAR.

To achieve this, artificial sterile tubes are connected to the arteries and veins of the biological organs, integrating them into the BAR. The heart (or its analogue) pumps a nutritious medium containing the necessary biological structures (e.g., red blood cells, platelets and blood proteins) inside these arteries, tubes and veins. The biological organs (or their analogues) required to maintain BAR homeostasis are the kidneys, liver, lungs, heart, etc. While BAR focuses on using chemical energy from glucose and fat oxidation, electrical components are still necessary for maintaining homeostasis, muscle contraction and data input. The force generated by the controlled, electronically induced biological muscle contractions inside the BAR is transferred to the robot skeleton, enabling locomotion. We believe that BAR is feasible as long as materials which contact with biological structures or nutritious medium, are biosafe, based on biohybrid robotics experiments (Morimoto et al., 2020; Ricotti and Menciassi, 2012; Morimoto et al., 2018). A rough draft of the BAR (Biohybrid Autonomous Robot) is displayed in Figure 1.

## 2 Methods

The primary aim of this article is to evaluate the BAR concept, yet due to the interdisciplinary nature of the BAR, the calculations performed are based on existing data from various fields. Therefore, it should be noted that no statistical methods were employed in the data analysis due to the heterogeneous nature of the data. Consequently, the calculated results should only be used as preliminary estimates of potential values. It is also important to recognise the limitations imposed by the assumptions made to simplify the calculations. All calculations in details and references to the data sources used can be found in the attached Supplementary Datasheet.

## 3 Study assumptions

The BAR concept and methods are explained, so the study’s assumptions must be stated. As we mentioned earlier, the BAR requires organs, for example, kidneys, liver, heart etc. We believe that for the rough estimation of the required organs’ mass–the human body composition may be used. In the Table 1 below we placed the mass of required organs and systems for BAR in absolute and relative numbers.

**TABLE 1:** The mass of the required organs and systems for BAR.

No	Organs and systems in the BAR	Absolute mass in the human body, kg	Relative mass in the BAR, %
1	Electronics, step motors and other electricity-powered devices (2 brain masses of the human body)	1.609 (Müller et al., 2011)	3.80%
2	Heart	0.384 (Müller et al., 2011)	0.91%
3	Liver	1.774 (Müller et al., 2011)	4.19%
4	Kidneys	0.329 (Müller et al., 2011)	0.78%
5	Fittings, tubes and adapters	—	1.00%
6	Lungs (assumption)	1.5	3.55%
7	Blood	—	8.00% (Oberholzer et al., 2024)
8	Containers for storing substrate	—	5.00%
9	Mass required for sustaining metabolism of the BAR (BAR’s Ballast mass)	—	27.23%
10	Non-ballast mass of the BAR	—	72.77%
11	Mass of human body	84.6 (Müller et al., 2011)	100.00%

According to the Table 1, 72,77% of BAR’s mass may be utilized to perform a locomotion, work and substrate storage, while 27,23% are a «ballast». Thus, in our calculation we used the mentioned 72,77% as a downgrading multiplier.

The calculations in the “Results” section are also based on the following assumptions: One Gram of glucose contains 4 kcal (4.65 Wh) of total stored energy (Unites, 2005). One Gram of fat contains 9.3 kcal (10.82 Wh) of total stored energy (Unites, 2005). The efficiency of electric motors is 83% (average of two values found) (Demirel, 2018). The densities of glucose and fat are 1.5 g/cm^3^ and 0.9 g/cm^3^, respectively (Sanjay, 2019; Wagner and Heyward, 2000). The molar masses of oxygen, glucose and palmitic acid are 32 g/mol, 180 g/mol and 256 g/mol, respectively (National Center for Biotechnology Information, 2025a; National Center for Biotechnology Information, 2025b; National Center for Biotechnology Information, 2025c).

The energy expenditure for the transportation of glucose, oxygen and fat (palmitic acid) into the cells is included into the basal metabolism of living system. We made that assumption due to the fact, that glucose and fat are transported via protein channels without energy consumption (due to concentration gradient of glucose and fatty acids within and around the cells) (Betts et al., 2022a). The overall efficiency of glucose and palmitic acid utilization in mitochondria, including all of the biochemical catabolic reactions can be considered to be not higher than 41% (Schmitz, 2012a) and 42% (Schmitz, 2012b) respectively, though some studies suggest lower efficiency of the mitochondria (Rich, 2003). In any case, the inefficiency of muscle contractions reduces the total efficiency to 20% (Smith et al., 2005).

The complete oxidation equation of glucose is used to calculate the energy capacity of the BAR when running on glucose and oxygen from a gas cylinder (National Center for Biotechnology Information, 2025a; National Center for Biotechnology Information, 2025b).

The complete oxidation equation of palmitic acid is used to calculate the energy capacity of the BAR when operating on fat and oxygen from a gas cylinder (National Center for Biotechnology Information, 2025a; National Center for Biotechnology Information, 2025c).

It is also assumed that biological muscles use either glucose or fat as an energy substrate in order to determine the upper and lower limits of the possible energy range of the Biohybrid Autonomous Robots (BAR). In reality, different muscles use both glucose and fat, though in different proportions in conditions if both are available.

## 4 Results

### 4.1 Results of calculations and arguments for the BAR concept

With the assumptions above stated, the calculations of the energy densities of the energy substrates of Biohybrid Autonomous Robots (BAR) and Conventional Autonomous Robots (CAR) were performed according to the Equation 1:

Uusef=U*OAE*ARnbm, (1)

where U–is energy density of the substrate, ARnbm–non-ballast-mass of the Autonomous Robot in %, OAE–overall actuator efficiency in %, U_usef_–useful energy density of the substrate.

**FIGURE 2:**
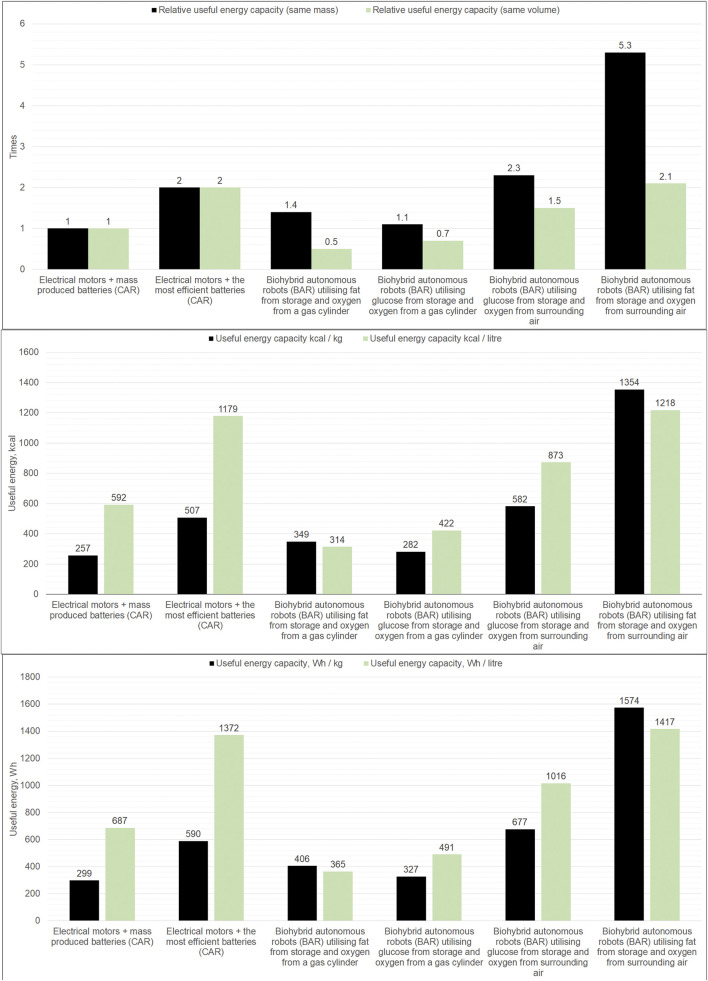
Total useful energy of batteries and electric motors in the CAR compared to the useful energy of utilisation of glucose, fat and oxygen in BAR, expressed in absolute and relative values.

Figure 2 show the results of the energy calculations for the Biohybrid Autonomous Robots (BAR) without the basal metabolism rates compared to (Conventional Autonomous Robots) CAR. A detailed description of the calculations can be found in a Supplementary Datasheet. Five conclusions are derived from the energy calculations.

1. Due to the high mass of oxygen required, it is not efficient to use gas cylinders to store oxygen inside the BAR; it is more rational to use oxygen from the atmosphere.
2. Glucose carries up to 2 times more useful energy than the mass-produced batteries of the same mass when atmospheric oxygen is used.
3. Glucose carries up to 1.4 times more useful energy than the mass-produced batteries of the same volume when using atmospheric oxygen.
4. Fat carries up to 5.1 times more useful energy than the mass-produced batteries of the same mass when using atmospheric oxygen.
5. Fat carries up to 2 times more useful energy than the mass-produced batteries of the same volume when using atmospheric oxygen.

Yet we did not discuss the basal metabolic rates of the biological parts in Biohybrid Autonomous Robots (BAR). It is impossible to tell a definite number without the model, so we created one to calculate the point when the BAR becomes viable alternative from the energy point to the CAR. The model can be found in the Supplementary Datasheet.

The main equation of the model is Equation 2:

$$\frac{BARbm+\left(\frac{UWP}{BAReff*BARum}\right)}{Ubar}<\frac{\left(\frac{UWP}{CAReff}\right)}{Ucar},$$ (2)

Where BARbm–basal metabolism of the BAR, UWP - useful work performed, BAReff - efficiency of the BAR, BARum–BAR useful (non-ballast) mass, CAReff - efficiency of the CAR, Ubar–energy density of the substrate inside the BAR, Ucar–energy density of the batteries inside the CAR. If the left side of the Equation 2 is smaller than the right side, than it is more rational to use BAR instead of the CAR from the energetics perspective.

According to our calculations, if the basal metabolic rate of BAR is 2,500 kcal per day, the BAR becomes an alternative in energetic sense to CAR in case if the amount of useful work performed is 230 kcal (267 Wh) for fat-powered BAR and 2,500 (2,908 Wh) kcal for glucose-powered BAR. In absolute numbers, with metabolism of the BAR included, the total energy used skyrockets to 4,080 kcal (4,743 Wh) for fat-powered BAR and 19,667 kcal (22,879 Wh) for glucose-powered BAR, which is approximately 0.43 kg of fat and 4.92 kg of glucose respectively. While we find it feasible that fat-powered BAR might be superior in terms of useful energy density of the substrate to the Conventional Autonomous Robots (CAR) with the most efficient batteries and thus implementable as an alternative, yet the glucose mass-to-carried energy proportions inside the BAR seem to not be sufficient to be an alternative to the CAR with the most efficient batteries found.

Also, there is an option to decrease the impact of basal metabolism on maintenance frequency of the BAR during periods of inactivity, if the BAR is stored at a low positive temperature (approximately 3 °C–5 °C) (Black et al., 1976).

Based on the assumptions, calculations and remarks presented above, it can be concluded that the Biohybrid Autonomous Robots (BAR) powered by fat currently has a theoretical energy advantage over the conventional autonomous robots (CAR), from some reasonable level of energy expenditure.

However, there are 2 points left undiscussed:

1. The practical utility of advantage of fat-powered BAR over CAR depends on other properties of biological muscles, such as strength and power generated per 1 cm^2^, the ability to control their motion precisely and the density of the biological structures.
2. There are theoretical alternatives to the Conventional Autonomous Robots (CAR) such as Generator-Powered Autonomous Robots (GPAR) and Fuel-Cell Autonomous Robots (FCAR).

We will start with the biological muscle properties. Biological muscles have been shown to have a decent force-to-mass and power ratio (specific values stated below) (Ricotti and Menciassi, 2012), good movement control and repeatability (Ricotti and Menciassi, 2012), regenerative and adaptive capabilities (Musarò, 2014).

**FIGURE 3:**
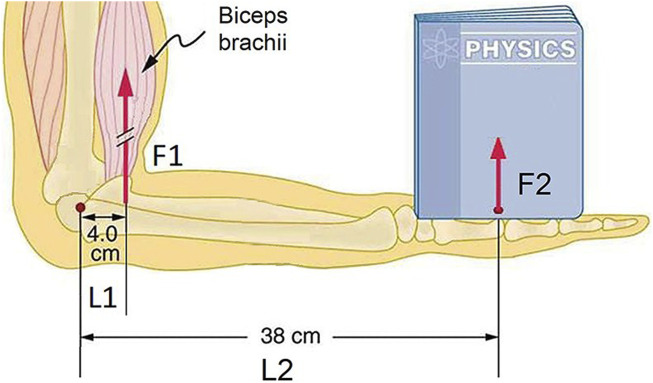
Model of the joint in the BAR and human body, adapted from (Hamm, 2020), which was used for calculations.

To further investigate the subject, we performed calculations, based on muscle model, which is illustrated in the Figure 3, and third type lever equation, shown in Equation 3; (Dickson, 2023).

F1*L1=F2*L2=Fcsa*CSA*L2=M1=M2=T, (3)

where F1 – force on the proximal end to the junction, L1 – is a length to the point of force F1 application, F2 – force on the distal end of the lever, L2 – the distance to the point of force F2 application, Fcsa–force per cross-sectional area of muscle, CSA–cross-sectional area of muscle, M1 – moment of the force 1, M2 – moment of the force 2, T–torque in the joint.

The results indicate that 1 cm^2^ of the cross-sectional area of biological muscles can generate forces ranging from 37 kg to 146.3 kg (calculations are included in the Supplementary Datasheet) (Dickson, 2023; Kashiri et al., 2018; Hamm, 2020; Ikai and Fukunaga, 1970; Ikegawa et al., 2008). The power generated per 1 cm^2^ of cross-sectional ranges from 13,7 W/cm^2^ to 54,1 W/cm^2^ (Conversion-calculator for measurement units, 2008). To give example of the given values, the 9.24 cm^2^ of biological muscle, which is a mean of biceps brachii of healthy men from study (Li et al., 2020), can produce force ranging from 342 kg to 1,349 kg without the joint and 36 kg–142 kg in the joint, while the power generated by muscle (biceps brachii) ranges from 127 W to 500 W. The differences in force of biological muscles per 1 cm^2^ could be explained by the various physiological, anatomical and other properties of different muscles (Hamm, 2020; Ikegawa et al., 2008; Betts et al., 2022b).

We labeled the power density of the biological muscles as “decent” above, yet we did not provide the power density of the electrical engines as context. The power density of the electric motor actuator may have a value of 170–500 W/kg (Kashiri et al., 2018). There is study that states that the power density of biological muscles is 40–225 W/kg, with the peak power of 1,000 W/kg (Ricotti and Menciassi, 2012). Our calculations, as we have already noted, also show that the power per 1 cm^2^ of biological muscle ranges from 13.7 W/cm^2^ to 54.1 W/cm^2^. Thus, we think that BAR may be inferior in terms of the regular actuation power density compared to the CAR in the case of the low cross-sectional area of biological muscle, yet in case if the mass of electric actuators and biological muscle is the same, BAR may be superior in the terms of the peak power density produced.

The last favourable point of the BAR is the low density as muscles have a density of 1.06 g/cm^3^ (Leonard et al., 2021), while the whole human body (taken as example due to the close supposed density to the average density of the biological part of the BAR) have a density of 1.096 g/cm^3^ (Heymsfield et al., 1989). Electronics, storage vessels for energy substrate and other non-biological parts will make the BAR denser, yet we doubt that it will come close to at least 2 g/cm^3^, thus making it advantageous compared to the Conventional Autonomous Robots (CAR), Generator-Powered Autonomous Robots (GPAR), Fuel-Cell Autonomous Robots (FCAR) with electric engines, yet not to other theoretical low-density actuator types (Aubin et al., 2022).

When we combine the findings above, BAR emerges as a potential alternative to the CAR, yet there are other alternatives, which we need to discuss.

Furthermore, the following sections will show that there are ethical and technical issues that arise during the development of BAR.

### 4.2 Alternatives to both BAR and conventional electric motor-battery model

We should say that there are a lot of combinations of actuators and energy-storing structures, according to the study (Aubin et al., 2022), yet as current study focuses on the solving the energy autonomy problem, we would like to highlight 2 alternatives to BAR and CAR – Generator-Powered Autonomous Robots (GPAR) and Fuel-Cells Autonomous Robots (FCAR), which use electric engines as actuators.

#### 4.2.1 Generator-powered autonomous robots (GPAR)

The generator systems are widespread, relatively easy to operate, their production is streamlined. The efficiency of the natural gas combined with combined cycle gas turbines is over 50%, for example, (taken as example due to the highest efficiency) (Khatib, 2012). Thus, adding the electrical engines efficiency (83%) (Demirel, 2018), the overall actuation efficiency of the GPAR is 41.5% if we suppose 100% efficiency of battery charge. The GPAR concept has its problems: temperature isolation, toxic combustion products, lack of the required small-sized versions, vibrations. Temperature isolation increases the technical difficulty of constructing and exploiting the robot, omitting the size of the system. The toxic products of the combustion CO, NO, etc. might make it unsafe to use those robots in the buildings.

Yet it is possible to create some hybrid between the conventional electric motor-battery model and the generator model, though robot will have a requirement to charge its batteries via combustion outdoors periodically. Though, there will be also a problem of ballast mass and space, as with the BAR, due to the required space and mass for generator, though the amount of required volume and mass depend on the construction Generator-Powered Autonomous Robot (GPAR). In calculations we assumed, that 40% of the (Generator-Powered Autonomous Robot) GPAR volume and mass can be considered as a ballast due to the necessity for enclosed self-sufficient reliable generator system. The details of the calculations below could be found in the Supplementary Datasheet.

The calculations were performed according to the Equation 1:

Uusef=U*OAE*ARnbm,

where U–is energy density of the substrate, ARnbm–non-ballast-mass of the Autonomous Robot in %, OAE–overall actuator efficiency in %, U_usef_–useful energy density of the substrate.

The useful energy density of natural gas according to calculations is estimated as 2,698 kcal/kg (3,138 Wh/kg) and 1,214 kcal/L (1,412 Wh/L) (Unites, 2005; The Engineering ToolBox, 2005).

#### 4.2.2 The fuel-cell system autonomous robots (FCAR)

Fuel cell systems represent an interesting alternative to BARs, conventional autonomous robots (CARs), and generator-powered autonomous robots (GPARs) due to their ability to directly generate electricity. Their efficiency ranges from 40% to 80% (Salameh, 2014). Yet, the fuel cells have the temperature isolation problem as an energy autonomy problem solution as fuel-cells require a temperature of more than 80 °C, up to 800 °C, depending on the fuel cell type, which makes it hard to apply the high-temperature fuel-cells, limiting the choice of possible fuel-cells (Salameh, 2014).

We suggest paying attention to the Direct Ethanol Fuel Cells (DEFC) as a power source for Fuel-Cell Autonomous Robots (FCAR). Their working temperature is 120 °C (Bishnoi et al., 2024), which is somewhat acceptable from the engineering point, though it raises practical technical questions, such as – where to dissipate the heat and how to do it efficiently. Ethanol, used in DEFC is safe for human and the energy density of DEFC theoretically is 6,879 kcal/kg (8,000 Wh/kg). Currently, Direct Ethanol Fuel Cells have the efficiency of 35%–60%. In the following calculations we assumed the efficiency of 48% as a mean of given values. Including the efficiency of electrical engines (83%) (Demirel, 2018), overall efficiency of DEFC-powered autonomous robot drops to 39.4%.

The calculations were performed according to the Equation 1:

Uusef=U*OAE*ARnbm,

where U–is energy density of the substrate, ARnbm–non-ballast-mass of the Autonomous Robot in %, OAE–overall actuator efficiency in %, U_usef_–useful energy density of the substrate.

According according to our calculations, the energy density of 1 kg of DEFC is 2,712 kcal/kg (3,154 Wh/kg). The details of the calculations could be found in the Supplementary Datasheet.

Thus, the Ethanol Fuel-Cells Autonomous Robots (EFCAR) may be feasible alternative concept to BAR, GPAR and CAR, though further research is required.

**FIGURE 4:**
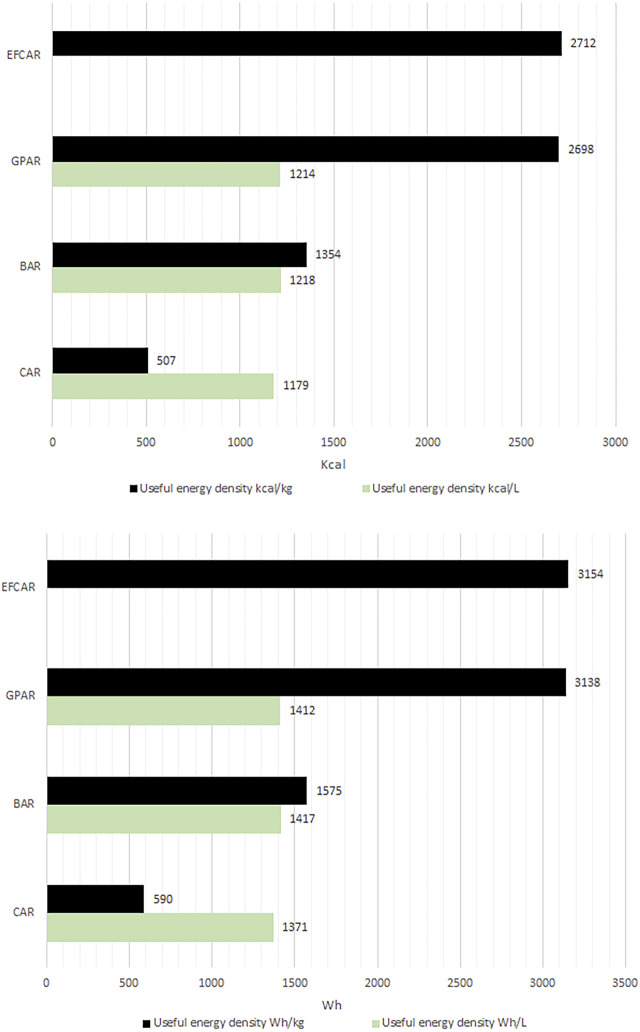
Comparing the energy densities of the substrates of CAR, BAR, GPAR, EFCAR taking into the account “ballast” mass and volume.

Also, to make the comparison of the BAR, GPAR, CAR and EFCAR easier, we created the Figure 4.

### 4.3 Ethical challenge of the BAR

After discussing the advantageous properties of the BAR and the alternatives, we shall discuss the problems of the concept. The ethical challenge arises since the BAR requires biological structures or their analogues, including muscles, tendons, bones, livers, kidneys, bladders, lungs, hearts, red blood cells, platelets and blood plasma without immune components. Some of the aforementioned structures can only be obtained from animals due to the lack of non-electrical alternatives, as well as the vascularisation problems associated with bioprinted organs (Später et al., 2020) (the ethically problematic biological structures required for the BAR and their methods of their acquisition are described in the 'Technical challenges of the BAR’ section in details). Thus, the BAR raises ethical concerns as it is not a life-saving necessity for humans, yet it is associated with significant animal suffering (Persson et al., 2025), which limits BAR industrial application with current technology, though in the perspective with bioprinting advances, the model loses its ethical troublesomeness.

### 4.4 Technical challenges of the BAR and their solutions

The question-and-answer format below outlines the technical challenges of the Biohybrid Autonomous Robots (BAR) and potential solutions.

#### 4.4.1 If the electrical parts are still required for the BAR, what is the point of it as an alternative to the conventional electric motor-battery models, such as CAR, GPAR and EFCAR?

The point is that low-power electronics use little power. While it is not possible to eliminate their use entirely due to technical constraints, we think that over 90% of the energy required for BAR operation can be derived from fat oxidation within the BAR itself. Furthermore, integrating mechanisms that use the energy produced by BAR motion to generate electrical power for its electronics is a feasible prospect.

#### 4.4.2 How can the biological structures within the BAR be maintained over the long term?

We believe, based on experiments in the field of biohybrid robotics (Morimoto et al., 2020; Ricotti and Menciassi, 2012; Morimoto et al., 2018), that BAR is feasible provided that the materials in contact with biological structures and the nutrient medium are biosafe. Therefore, we will consider the conditions necessary to ensure the long-term viability of BAR:

1. Biological structures are connected to each other by biosafe sterile tubes. 2. A blood analogue consisting of a nutrient medium, red blood cells, thrombocytes, hormones, antimicrobial agents and blood plasma proteins without immune components circulates inside the blood vessels and artificial tubes via the heart pumping or its analogue. 3. The kidneys or their analogues filter the blood. 4. The liver or its analogue performs the biochemical reactions necessary for BAR functions, synthesising blood plasma proteins (components of the blood coagulation and anticoagulation systems, and fibrinolysis components and blood transport proteins), recycling erythrocytes and platelets. 5. The lungs or their analogues perform gas exchange. 6. Hormones regulate organs’ functions. 7. Aseptic conditions are maintained within the system (a process facilitated by antimicrobial agents and the design of the BAR). 8. The introduction of nutrients and hormones is uncomplicated. 9. The bladder or its analogue stores waste products. 10. The removal of waste products is uncomplicated. 11. Temperature and chemical homeostasis are ensured. 12. The correct balance between rest and work is ensured. 13. Biological muscles are stimulated by repetitive electrical signals. 14. Muscles have antagonists in the joints, as it has been observed that biological muscles shrink in the absence of antagonists (Morimoto et al., 2018).

#### 4.4.3 How can biological muscles and other biological structures be obtained for the BAR?

The biological structures (or their analogues) required for the BAR, mentioned in the 'Ethical Challenge of the BAR’ section, are listed in order from the most to the least ethically problematic from the perspective of the acquisition methods.

Muscles, tendons and bones are the most ethically problematic structures. Currently, these organs or parts of them must be surgically removed from the donor animal and the animal must be euthanised. There are two reasons for this: firstly, there is a natural, continuous chain of force transmission from muscle to tendon to bone (Gaut and Duprez, 2016), which cannot be artificially constructed due to the poor mechanical properties of artificial tendons (Moslem and Shafaie, 2025); secondly, bioprinted organs have a vascularisation problem (Später et al., 2020), which makes bioprinting not feasible solution.

The second group of biological structures consists of the liver, kidneys, lungs and heart. As with the first group of organs, it is preferable to harvest these organs from animals and then euthanise them. However, existing technologies can be used to produce inferior artificial alternatives that do not require euthanising animals, which can be useful during early-stage experiments and to alleviate public repulsion to the experiments on animals for the BAR development.

The third group consists of red blood cells, thrombocytes and blood plasma without immune components (e.g., blood transport proteins, proteins of the coagulation, anticoagulation and fibrinolysis systems). Procuring erythrocytes, platelets and blood plasma without immune components from donor animals does not raise significant ethical concerns.

The fourth group does not raise ethical concerns as it consists of hormones, bladder and the immune system. In theory, the bladder could be bioprinted (Chae et al., 2022) or replaced with an artificial analogue to store the waste products of BAR. Hormones could be artificially synthesised (Roytrakul et al., 2001). Regarding the immune system, we believe that it should not be included into the BAR due to the numerous limitations associated with its response to foreign organs and artificial constructs (Moreau et al., 2013; Ishai et al., 2017). While recognising the importance of immune system functions as a defence mechanism, we propose suppressing the immune system and replacing it with antimicrobial agents and design features to maintain aseptic conditions during operation and maintenance.

#### 4.4.4 How can muscle contractions be controlled?

*In vitro* biohybrid experiments electrodes are used to control muscle tissue (Morimoto et al., 2020; Ricotti and Menciassi, 2012; Morimoto et al., 2018). We could apply this system of electrodes in the BAR, though there is a risk that the electrodes inside the BAR may become encapsulated in fibrous tissue over time, as occurs *in vivo* (Ishai et al., 2017). However, the situation is unclear due to the presence of immunosuppressive drugs and the absence of most of the immune organs within the BAR. Due to that, the electrodes may not become encapsulated, as the immune system plays an important role in fibrosis (Jones, 2020). In any case, for the BAR to function, the electrodes must be able to stimulate each muscle in order to enable controlled contraction and maintain muscle homeostasis.

#### 4.4.5 How can BAR temperature homeostasis be maintained at different levels of physical activity?

It is hypothesised that the BAR includes the biological muscles and structures of warm-blooded animals. The crux of the problem is that the optimal operating temperatures of the biological structures of warm-blooded animals are limited, whereas the BAR’s heat generation is significantly influenced by the level of activity. We assume that the target temperature of biological muscles inside the BAR is 37 °C and the deviations of less than 1 °C from this temperature are acceptable, based on the fact that the optimal temperature for human muscles to perform the work is 36 °C–38 °C (Inoue et al., 2014) (the human body is used as an example due to the abundance of data available). To illustrate the problem more vividly, we made five assumptions to create a hypothetical example.

1. Men and men-sized BAR have the same metabolic rate, the same efficiency of resting metabolic rate, when performing equivalent physical activities. Men are used as a reference point because they have a higher proportion of muscles than women.
2. The average resting metabolic rate for men is 84.3 W (72.5 kcal per hour) (Unites, 2005; Arciero et al., 1993).
3. The efficiency of resting metabolic rate of all humans, men included, is approximately 25% (assumption).
4. The total additional energy expenditure during the intensive work period is 1,000 kcal per hour (1,163 Wh).
5. Biological muscles have an efficiency of 20% (Smith et al., 2005), meaning that 80% of the additional 1,000 kcal (1,163 Wh) of energy consumed by BAR during the intensive work period, is converted into heat. Details of the calculations below can be found in the Supplementary Datasheet.

Calculations were performed according to the Equation 4:

Nrel=NintworkNrest, (4)

where N_rel_–relation of the heat power produced during intensive work and rest, N_int_work_–heat power produced during intensive work, N_rest_–heat power produced during rest.

The difference in heat production between rest and intense work is 15.7-fold, according to the results of calculations. Given these variations in heat production, maintaining a target temperature and acceptable temperature deviations poses a technological challenge. However, the human body has developed mechanisms to address this issue, such as sweating and constricting and dilating blood vessels, which could be borrowed.

#### 4.4.6 How can the force be transferred from the biological muscle to the robot?

There is a natural, continuous chain of force transmission from muscle to tendon to bone (Gaut and Duprez, 2016). This chain can be modified to suit the force transmission objectives of the BAR. To achieve this, it is sufficient to attach an artificial clamp to a biological bone within this chain. Provided the clamp is biosafe and sufficiently robust, the force generated by the biological muscle can be transmitted to the robot via the tendon, bone and clamp, and then onto the robot skeleton (see Figure 1).

#### 4.4.7 Will the process of production and maintenance be expensive?

We do not have a good answer to this question, due to scale effect, and due to the further research required. It can be argued that small-scale production and maintenance of BAR will lead to high costs. Large-scale production could allow for the production and maintenance of BAR at reasonable prices.

### 4.5 The comparison of the CAR, BAR, GPAR and EFCAR

To make the results of the study clearer we decided to create a Table 2, in which we describe the main strengths and weaknesses of the discussed models.

**TABLE 2:** The overall comparison of the CAR, BAR, GPAR and EFCAR.

Model	Useful energy density per liter, kcal/liter (Wh/liter)	Useful energy density per kg, kcal/kg (Wh/kg)	Strength	Weaknesses	Overall actuator efficiency	Ballast mass
CAR with the best batteries found	1,179	507	1. Most developed field 2. Easy to maintain	1. Low energy density of the batteries	83%	0%
BAR	1,218	1,354	1. Low density of the system 2. High peak power density and decent regular power density of biological muscles 3. Loses mass during the work 4. Regeneration and adaptation ability	1. High risk of contamination in case of the protocols breach 2. Requires further research	20%	27,33%
GPAR	1,214	2,698	1. The fuel is inexpensive 2. Loses mass during the work	1. Requires outdoor charging of the batteries	41.5%	40%
EFCAR	—	2,712	1. The fuel is inexpensive 2. Loses mass during the work	1. Generate high temperature during the work	39.4%	0%

## 5 Discussion

In principle, it is possible to manipulate artificial objects using biological structures in robots, as demonstrated in previous experiments (Morimoto et al., 2020; Ricotti and Menciassi, 2012; Morimoto et al., 2018). However, we have not found any study analyzing the feasibility of implementing biohybrid robots and putting it into the context of the alternative solutions of the energy autonomy problem of the autonomous robots. We would like to remind our readers that the main objective of the paper was to analyze the BAR concept in detail. The results of the calculations can only serve as preliminary estimates of the energy values of the BAR, CAR, GPAR, EFCAR, force and power per 1 cm^2^ of biological muscle cross-sectional area. In our opinion, the BAR concept has advantages, such as the high useful energy density of fat, theoretical low density of the BAR and biological muscle properties, as well as challenges, such as construction requirements and ethical issues. We believe that BAR could be considered on par with CAR, GPAR and EFCAR as a way to achieve energy autonomy of robots, although its implementation raises many challenges. As this is a theoretical paper, many experiments are required to either support or reject the concept.

## 6 Conclusion

The potential of the BAR lies in:

1. The decent force generated by biological muscles per 1 cm^2^ of cross-sectional area (37.5–146.3 kg).
2. The decent power of biological muscles per 1 cm^2^ (13.7 W/cm^2^ to 54.1 W/cm^2^), decent regular actuator power density (40–225 W/kg), a high peak power density (1000 W/kg).
3. Low density of the energy substrates and of the BAR itself (we suppose that density of the BAR will be lower than 2 g/cm^3^).
4. Competitive level of useful energy of the fat-powered BAR per unit of mass and volume of energy substrate (1,218 kcal/L (1,417 Wh/L) and 1,354 kcal/kg (1,575 Wh/L)), compared to the CAR, GPAR, EFCAR.
5. The BAR is independent from the electrical grid as required energy for work is produced from the chemical source of power in the BAR itself.

Potential applications of BAR include:

1. Rescue operations in areas with limited access to electricity, such as earthquake zones. For example, in theory it would be possible to store 10 kg of fat in the BAR, which could provide an energy supply for 15 days of work without the need for recharging, with a total energy expenditure of 6,978 Wh (6,000 kcal) per day.
2. Creation of the fully controlled physiological model of the musculoskeletal system, which may consist of the bioprinted limbs trained by natural body movements performed during work. The trained bioprinted limbs may be used for the transplantation in case of limb loss.
3. Creation of the fully controllable model for overuse injuries in the development of rehabilitation protocols.
4. Obtaining a large amount of experimental data in the fields of immunology, cyborgization, bioengineering in the course of research, which allows for progress in these areas.

We anticipate that the results of this study will facilitate the development of the BAR as an alternative to the CAR, GPAR and EFCAR, as well as of the CAR, GPAR EFCAR themselves.

## Data Availability

The original contributions presented in the study are included in the article/Supplementary Material, further inquiries can be directed to the corresponding author.

## Abbreviations

Biohybrid robot, is a robot that uses biological structures to perform its functions.
